# Linear epitopes on the capsid protein of norovirus commonly elicit high antibody response among past-infected individuals

**DOI:** 10.1186/s12985-023-02087-y

**Published:** 2023-06-06

**Authors:** Yilin Deng, Taojun He, Bin Li, Hanmei Yuan, Fang Zhang, Hui Wu, Jie Ning, Yanping Zhang, Aixia Zhai, Chao Wu

**Affiliations:** Department of Laboratory Medicine, The Eighth Affiliated Hospital of Sun Yat-sen University, Shenzhen, 518033 Guangdong China

**Keywords:** Epitopes, IgG, Norovirus, VP1, VP2

## Abstract

**Background:**

Human norovirus (HuNoV) is the leading cause of acute nonbacterial gastroenteritis globally, and its infection is usually self-limited, so most people become past Norovirus (NoV)-infected individuals. It is known that some antibody responses may play a critical role in preventing viral infection and alleviating disease; however, the characteristics and functions of particular antibody responses in persons with previous infections are not fully understood. Capsid proteins, including VP1 and VP2, are crucial antigenic components of NoV and may regulate antibody immune responses, while epitope-specific antibody responses to capsid proteins have not been comprehensively characterized.

**Methods:**

We prepared purified VP1 and VP2 proteins by ion exchange chromatography and measured serum antigen-specific IgG levels in 398 individuals by ELISA. Overlapping 18-mer peptides covering the full length of VP1 and VP2 were synthesized, and then we identified linear antigenic epitopes from 20 subjects with strong IgG positivity. Subsequently, specific antibody responses to these epitopes were validated in 185 past infected individuals, and the conservation of epitopes was analyzed. Finally, we obtained epitope-specific antiserum by immunizing mice and expressed virus-like particles (VLPs) in an insect expression system for a blockade antibody assay to evaluate the receptor-blocking ability of epitope-specific antibodies.

**Results:**

The IgG responses of VP1 were significantly stronger than those of VP2, both of which had high positive rates of over 80%. The overall positive rate of VP1-IgG and/or VP2-IgG was approximately 94%, which may be past NoV-infected individuals. Four linear antigenic B-cell epitopes of capsid proteins were identified, namely, VP1_199–216_, VP1_469–492_, VP2_97–120,_ and VP2_241–264_, all of which were conserved. The IgG response rates of the above epitopes in past NoV-infected individuals were 38.92%, 22.16%, 8.11% and 28.11%, respectively. In addition, VP1_199–216_- and VP1_469–492_-specific antibodies can partially block the binding of VLPs to the receptor histo-blood group antigen (HBGA).

**Conclusion:**

This is the first study to describe specific antibody responses of VP2 and to identify its B-cell epitopes. Our findings offer data for a more thorough understanding of norovirus capsid protein-specific IgG responses and could provide useful information for designing and developing vaccines.

**Supplementary Information:**

The online version contains supplementary material available at 10.1186/s12985-023-02087-y.

## Introduction

HuNoV, the main cause of acute nonbacterial gastroenteritis in all age groups, is one of the targets of surveillance for foodborne diseases [1, 2]. NoV is highly contagious, requiring only 18 virions to infect a host, and poses a significant burden to global health and the economy [3]. GII.4 has always been a dominant genotype in norovirus epidemacs, besides, recombinant GII.4 Sydney 2012 [P16] is the most recent pandemic strain causing significant burden worldwide and has attracted much attention. The main symptoms of NoV infection are acute diarrhea and vomiting, and the infections are usually self-limiting, so most people reach convalescence [4, 5].

The NoV genome is approximately 7.6 kb in length and consists of three open reading frames (ORFs), ORF1, 2, and 3, which encode non-structural proteins (NS), major capsid protein (VP1), and minor capsid protein (VP2), respectively [6]. VP1 can be divided into the conserved inner shell domain (S domain) and variable outer protruding domain (P domain), while the P domain is further divided into P1 and P2, and the P2 domain mediates the binding of norovirus to the receptor HBGA on the surface of host cells [7, 8]. Current vaccine candidates are mainly VLPs formed by the self-assembly of VP1, which is known to be the key to immunogenicity and receptor binding [9, 10]. VP2 is believed to regulate viral replication and the expression of antigen-presenting molecules, and it can be co-expressed with VP1 to increase the stability of VLPs [11–13]. Consequently, both capsid proteins are crucial antigenic components of NoV, and they likely modulate immune responses.

The antibody-mediated immune response is critical in viral clearance after infection, however, the specific antibody response to NoV has not been fully revealed. VP1-specific antibody responses have been thoroughly investigated, and a few papers have also suggested the existence of NS protein antibody responses; however, VP2-specific antibody responses have not yet been characterized [14]. Some specific antibody responses have been shown to prevent viral infection and alleviate disease, meanwhile, the antibody activation may be more durable than that of T cells [15]. In particular, IgGs constitute the majority of serum antibodies, and previous studies have indicated that the average prevalence of serum NoV-specific IgG in the population is approximately 90% [16]. Therefore, the specific IgG response to NoV is of great concern. Studies have shown that serum from the acute phase of NoV infection had a high titer of VP1-specific IgG, which was higher in the recovery phase (day 14 postinfection) and mostly seroconverted, and the titer remained high for up to 1 year postinfection. Blocking antibodies were almost undetectable in the acute phase, and seroconversion occurred in approximately 45% of patients in the recovery phase [17, 18]. Another study demonstrated that systemic IgA and IgG antibody responses were closely correlated with blocking antibody responses and showed seroconversion of IgA, IgG, and blocking antibodies in 80%, 78%, and 87% of patients, respectively, 21 days after NoV infection [19]. Moreover, Alvarado et al. revealed that monoclonal antibodies isolated from patients previously infected with norovirus have neutralizing activity [20]. Although the antibody responses after NoV infection have been partially characterized, they are still not fully defined.

There are no specific treatments or vaccines for NoV, while many candidate vaccines are still in the clinical trial stage [21, 22]. The study of protective antibody is vital in the design and development of vaccines. Many conformational B-cell epitopes and few linear epitopes on VP1 identified from humans have been reported, and most of them are located in the P domain, which is variable [23–28]. Antibodies targeting the HBGA binding sites can directly block VLP binding to the HBGA receptor, while targeting other sites can interfere with their binding through other indirect effects, such as steric hindrance or allosteric effects [29, 30]. Of these, blockade epitope A is regarded as immunodominant, and about 40% of serum antibody blockade responses target it, but it is highly variable [31]. In addition, some antigenic sites are immunodominant, such as sites 1–43, 361–403, 453–495 of VP1 targeted by multiple monoclonal antibodies [23, 32, 33]. Compared with the in-depth study of VP1, little is known about VP2, and the B-cell epitope of VP2 has not been reported so far [23]. Therefore, the epitope research of NoV needs to be further supplemented, and it is urgent to explore conservative functional epitopes.

In this study, we focused on past NoV-infected individuals to comprehensively reveal the serum-specific IgG responses to VP1 and VP2. At the same time, we discovered linear blockade epitopes on VP1 and first reported the linear B-cell epitopes of VP2. Our work provides new data for a comprehensive understanding of NoV capsid protein-specific IgG responses, aiming to complement norovirus-related protective mechanisms, which may lay a sound foundation for optimizing vaccine components and development strategies.

## Materials and methods

### Subjects and blood samples

Blood samples from a total of 398 subjects who had been examined at the Eighth Affiliated Hospital of Sun Yat-sen University since 2021 were collected (Additional file 1: Table S1), and the subjects’stool samples were confirmed to be negative by NoV-PCR to exclude current infection. After 20 min of centrifugation at 3500 rpm, serum samples were collected and sub-packaged at − 80°C.

### Proteins and peptides

The VP1 or VP2 sequence was constructed into the vector pET28a(+) based on the reported sequence of norovirus GII.4 Sydney 2012 [P16] (GenBank accession number: NC_039477) and expressed as inclusion bodies in *Escherichia coli* (BL21,DE3). Then, we used an AKTA pure system (GE Healthcare, USA) to purify VP1 and VP2 proteins with purity greater than 90% by ion exchange chromatography. The eluents were then enriched, and the buffer was replaced using an ultrafiltration tube (Millipore, Germany). Protein concentration and purity were assessed using a BCA kit and SDS‒PAGE electrophoresis (Additional file 1: Fig. S1A, B), respectively. Eighty-eight and forty-three synthetic overlapping peptides, which spanned the entire length of VP1 and VP2 (GenBank accession number: YP_009518842.1, YP_009518843.1) of norovirus, were constructed according to the known sequence and synthesized by China Peptide Co., Ltd. (Shanghai, China). The synthesis of peptides moves 6 amino acids at each step, resulting in a length of 18 amino acids with 12 overlapping amino acids. The purity of all the above peptides was expected to be 90% or higher. The same company also created the epitope peptide-conjugated keyhole limpet hemocyanin (KLH) and the negative control peptide OVA_323–339_ (ISQAVHAAHAEINEAGR).

### Enzyme-linked immunosorbent assay (ELISA)

For VP1- and VP2-specific IgG tests, 96-well ELISA plates (Thermo Fisher, USA) were coated with 2 μg/mL purified VP1 or VP2 protein at 4 °C overnight. Plates were blocked using 1% BSA in PBST with 0.05% Tween-20 for 2 h at 37 °C. Then, 1:100 diluted serum was added in PBST containing 0.5% BSA and incubated for 1 h at 37 °C, followed by incubation with horseradish peroxidase (HRP)-conjugated goat anti-human IgG (ZSGB-Bio, China) for 1 h at room temperature. PBST was used to wash between each step. Finally, 100 μL of TMB substrate solution (TIANGEN, China) was added for color development for 15 min, and then an equal volume stop solution (Sangon Biotech, China) was added to terminate the reactions. The absorbance was determined at 450 nm wavelength via a microplate spectrophotometer (Thermo Scientific Varioskan LUX, USA). For the peptide-specific IgG test, the procedure was similar to the one described above, except that the coated antigen was 5 M peptides, the serum dilution was 1:25, and the secondary antibody was diluted to 1:5000.

### Generation of VLPs

We constructed NoV-VP1 between the pFASTbac1 vector BamHI/SphI and successfully expressed virus-like particles in the insect Sf9 cell expression system. To purify the VLPs, the soluble fraction of sf9 cell lysate was subjected to discontinuous sucrose density gradient centrifugation. First, the VLPs were concentrated by ultracentrifugation (Beckman Optima XE-90, Type 70i) at 35,000 rpm for 2 h at 4 °C and overlaid on a 20% sucrose cushion. Then, VLPs were loaded onto a 20–60% discontinuous sucrose gradient and ultracentrifuged horizontally (Beckman Optima XE-90, SW 28 rotor) at 23,500 rpm for 16 h at 4 °C. The VLP band, which was visible in the 40% sucrose layer, was collected. Then, VLPs were concentrated by ultracentrifugation at 23,500 rpm for 2 h at 4 °C. High-purity VLPs were obtained by ion exchange chromatography using the Akta pure system. They were then treated with the phosphotungstic acid negative staining method and examined under a transmission electron microscope.

### Preparation of antiserum

Six- to eight-week-old SPF female BALB/c mice (n = 10 for each group) were immunized three times at two-week intervals, and each mouse was injected with adjuvant plus 100 μg peptide-KLH conjugations or 100 μL NaCl. The first injection utilized Freund's complete adjuvant; the second and third injections utilized Freund's incomplete adjuvant (Sigma‒Aldrich). One week after the last immunization, antisera were collected, and their titer was detected using indirect ELISA.

### VLP-carbohydrate binding antibody blockade assay

A surrogate blocking assay was used to measure neutralizing antibodies. Ninety-six-well plates were coated with 5 μg/mL pig gastric mucin (PGM) Type III (Sigma‒Aldrich) in PBS, incubated for 4 h at room temperature, and then blocked with 5% no-fat milk in PBST at 4 °C overnight. Serially diluted antiserum samples were mixed with an equal volume of 2 µg/mL VLPs and incubated for 1 h at 37 °C. One serum-free well was used as a control for calculating the blocking rate. Then, the mixtures were added to the PGM-coated plates and incubated for 1 h at 37 °C. After washing three times, the plates were incubated with rabbit anti-VLP antibody (Absolute antibody, UK) for 1 h at 37 °C, followed by incubation with HRP-conjugated anti-rabbit IgG for 1 h at room temperature. After color development, the absorbance was determined at 450 nm. The blocking rate was calculated as (mean OD_450_ treated without serum − mean OD_450_ treated with serum)/mean OD_450_ treated without serum × 100%.

### Sequence alignment and structural modeling

The VP1 and VP2 epitope sequences were compared with the epidemic norovirus strains using Weblogo 3 (http://weblogo.threeplusone.com/). The crystal structure of the VP1 monomer was obtained from the Protein Data Bank (PDB) with accession code 7K6V and then constructed by SWISS-MODEL and PyMOL.

### Data analysis

All OD_450_ values were subtracted from blank control values. With irrelevant protein BSA or peptide OVA as the negative control, its mean OD_450_ plus 3 times the standard deviation was calculated as the positive cut-off value. Two independent repeated experiments were performed for each sample. GraphPad Prism 9.0.0 software was used to perform the Kruskal‒Wallis test and chi-square test. The hypothesis test was two-sided, and the level of statistical significance was set at *P* < 0.05.

### Ethics statement

The research protocols for this study were approved by the Medical Research Ethics Committee of the Eighth Affiliated Hospital of Sun Yat-sen University (No. 2020-058-01).

## Results

### VP1- and VP2-specific IgG responses were present broadly in past NoV-infected individuals

**Fig. 1:**
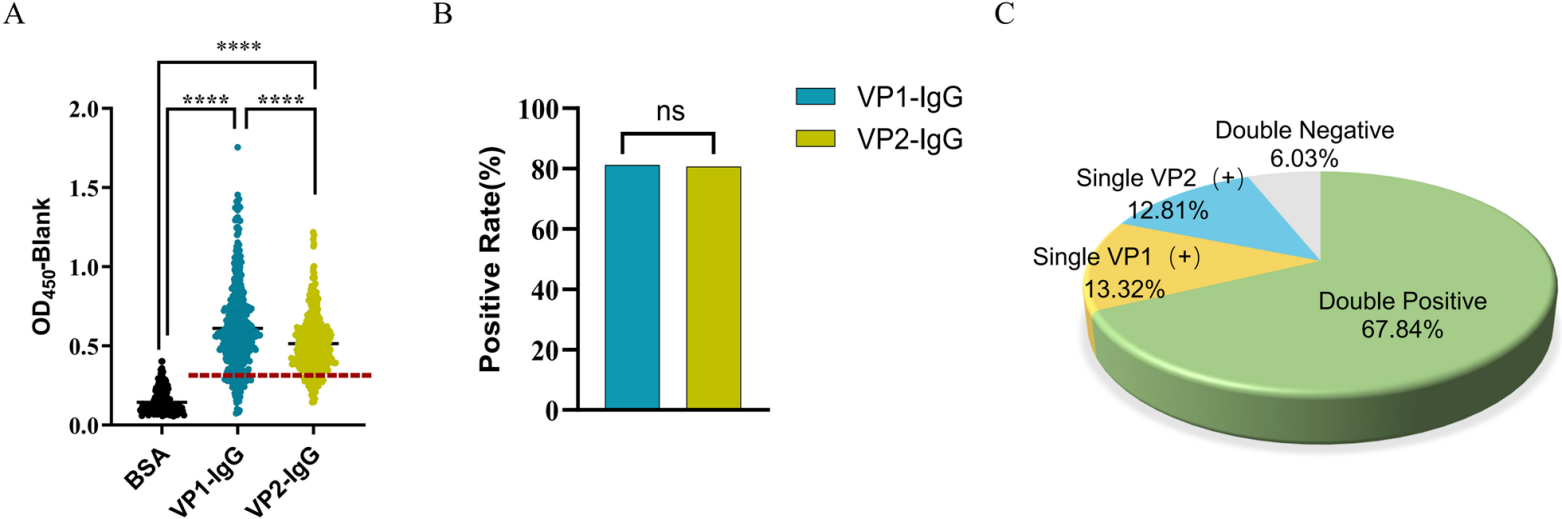
VP1- and VP2-specific IgG responses in non-current NoV-infected individuals. **A** The magnitude **B** and positive rate of VP1- and VP2-specific IgG responses. Serum samples were collected from 398 individuals who were not currently NoV-infected. All OD_450_ values were subtracted from blank control values. With irrelevant protein BSA as the negative control, its mean OD_450_ value plus 3 times the standard deviation was calculated as the positive cut-off value, and above the red dotted line was the positive response. **C** The results of 398 cases were classified. Statistically significant differences were determined by the Kruskal‒Wallis test and Chi-square test: ns, no significance; ****, *p* < *0.0001*

Serum VP1- or VP2-specific IgG in 398 individuals who were not currently NoV-infected was measured by ELISA. The magnitude of the VP1-specific IgG response was significantly greater than that of the VP2-specific IgG response (Fig. 1A); however, the positive rates of both were above 80%, with no significant difference (Fig. 1B). This indicates that both VP1 and VP2 antigens can generally activate specific IgG responses. There were no significant differences found in IgG responses between different sexes and age groups (Additional file 1: Fig. S2), which is a good reflection of the susceptibility to NoV. Correspondingly, we divided the results of all 398 cases into four categories, among which 67.84% of individuals were VP1- and VP2-specific IgG double positive, 13.32% and 12.81% individuals were VP1-IgG single positive, and VP2-IgG single positive, respectively. We considered the aforementioned individuals to have been infected with NoV in the past, and they represent approximately 94% of the population. The remaining 6.03% of individuals were negative for both VP1- and VP2-specific IgG and were considered to have never been infected with NoV (Fig. 1C). We hypothesized, above distributions may be related to exposure history, time of previous infection, and individual responses, but this requires further investigation.

### Capsid protein-specific IgG usually targets several linear epitopes

**Fig. 2:**
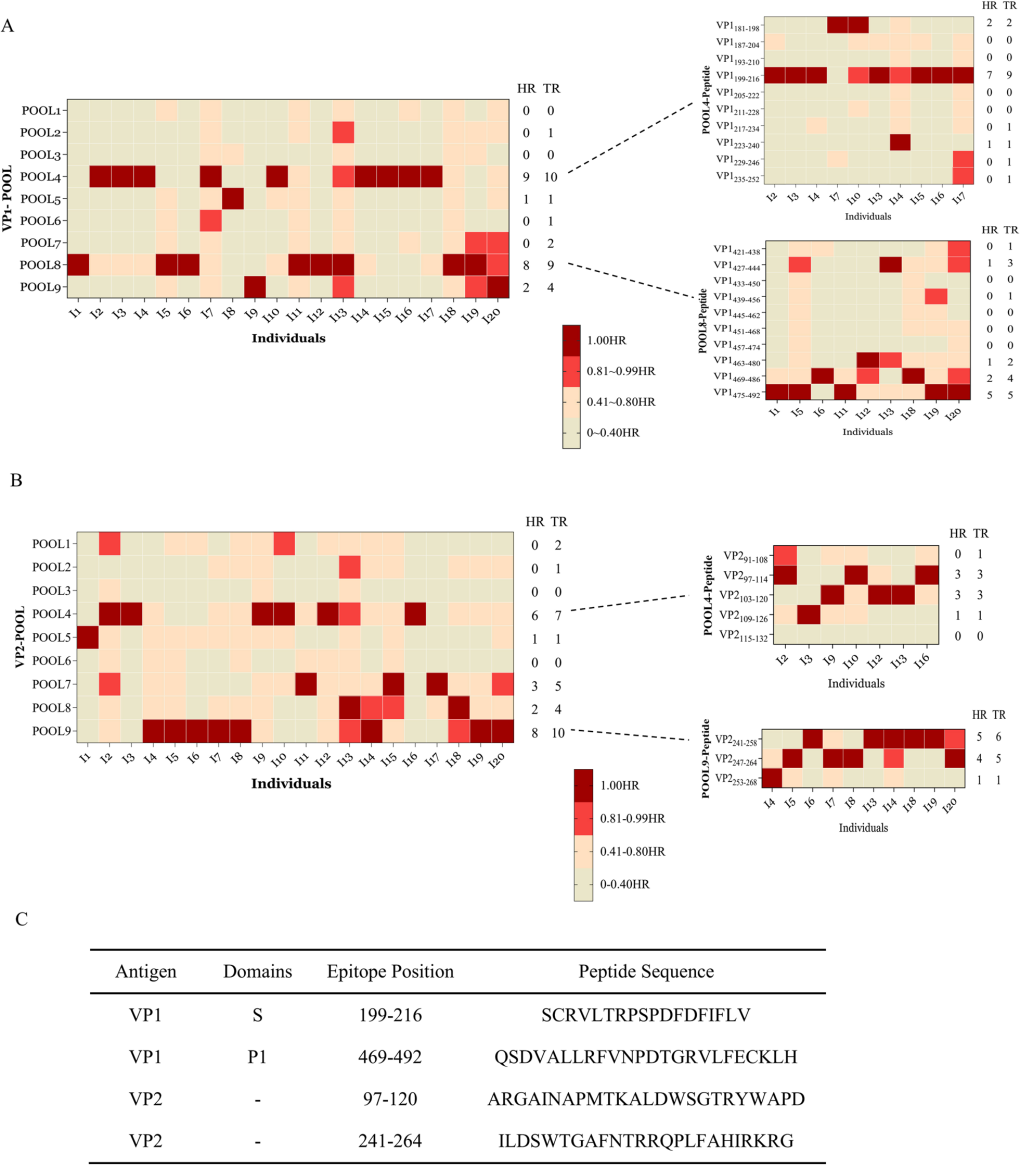
Mapping linear B-cell epitopes of VP1 and VP2. **A** VP1 and **B** VP2 of the heatmap for screening linear B-cell epitopes identified in 20 double-positive subjects. The individual's specific IgG responses to 9 matrix pools and corresponding peptides were detected. For each individual, the highest IgG response to one pool or peptide in a pool was noted as "1" and considered as high response (HR), filled with a dark red mark, the other responses were evaluated by their relative strength against HR. The responses with strength between 81 and 99% of the HR was considered as a sub-high response and was marked in red. Total responses (TR) included all highest and sub-high responses. **C** Domain, sites and amino acid sequence information of all linear epitopes

Since there are significant IgG responses to VP1 and VP2 in past NoV-infected individuals that may contribute to viral clearance, we attempted to search for antigenic B-cell epitopes from them. To determine the linear epitopes of VP1 and VP2, 88 and 43 18-mer overlapping peptides that spanned the full length of VP1 and VP2 were synthesized and divided into 9 matrix pools, respectively (see Additional file 1: Table S2, S3). From 20 cases of past NoV-infected individuals (serum VP1 and VP2 IgG strongly positive, with sufficient serum samples), using the peptide ELISA method, linear epitopes were identified by the screening strategy “from matrix pools to peptide”. As shown in Fig. 2A, the VP1-specific IgG response usually targets two regions, pool 4 and pool 8. Further measurement of individual peptides within pools narrowed the antigenic region to peptides VP1_199–216_ and VP1_469–486,475–492_. Similarly, two matrix pools from the VP2 library, pool 4 and pool 9, were frequently detected in serum from past NoV-infected individuals. Further, VP2_97–114, 103–120_ and VP2_241–258, 247–264_ were identified as linear antigenic B-cell epitopes in the above pools (Fig. 2B). As a result, capsid protein-specific IgG usually targets four linear antigenic epitopes in past NoV-infected individuals, namely, VP1_199–216,_ VP1_469–492,_ VP2_97–120_ and VP2_241–264._

### Linear epitopes elicit IgG responses universally in past NoV-infected individuals

**Fig. 3:**
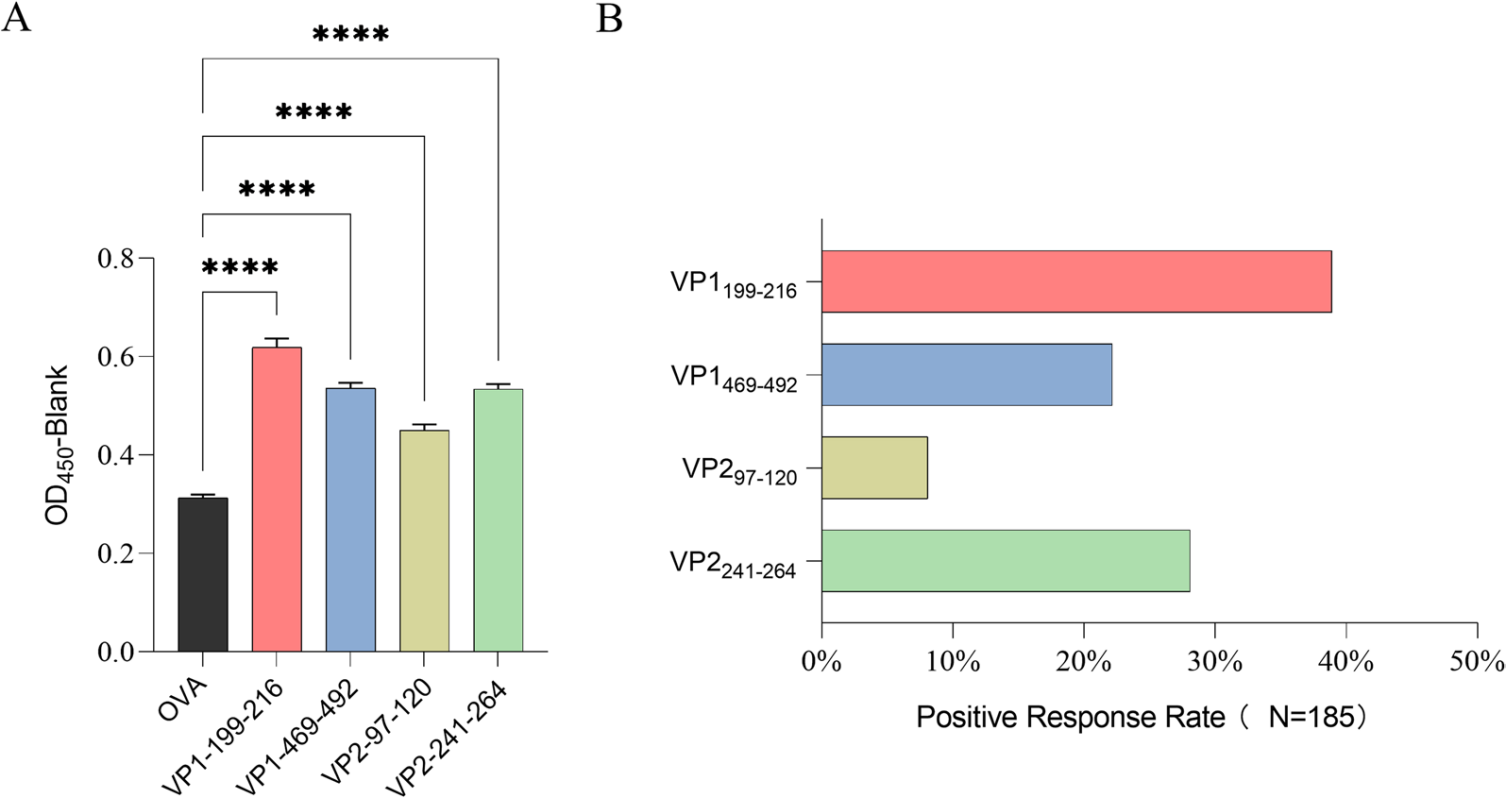
The IgG responses of epitopes were detected in more populations. **A** Specific IgG levels and **B** positive rates of each linear epitope were detected in 185 individuals. Irrelevant peptide OVA was used as a negative control, and its mean OD_450_ plus 3 times the standard deviation was used as the positive cut-off value to calculate the positive rate. Statistically significant differences were determined by the Kruskal‒Wallis test: ****, *p* < *0.0001*

To explore the general IgG responses elicited by the above linear epitopes in past-infected people, we measured the level of epitope-specific IgG among 185 individuals who were VP1 and VP2 IgG double positive. The level of each epitope-specific IgG was significantly higher than that of OVA, suggesting that the IgG responses are specific and powerful (Fig. 3A). A total of 38.92% of people present a specific IgG response to VP1_199–216_ epitopes, which also activates the highest level of IgG. The next epitopes were VP1_469–492_, VP2_97–120,_ and VP2_241–264_, and their IgG positive rates were 28.11%, 22.16%, and 8.11%, respectively (Fig. 3B). In conclusion, we further confirmed the universality of these epitopes in eliciting IgG responses.

### The linear epitopes showed good conservation among common strains

**Fig. 4:**
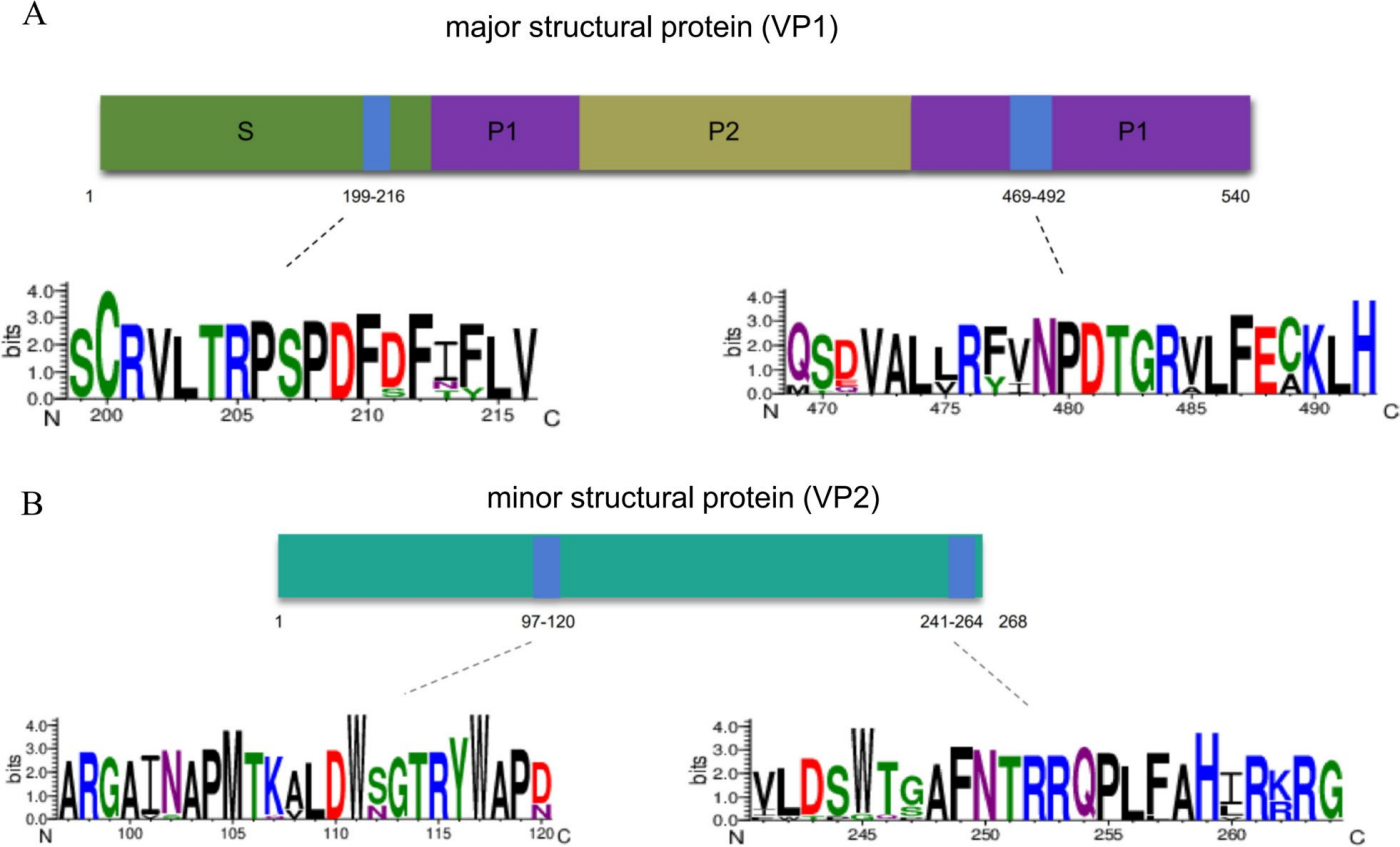
Sequence similarity of epitopes among common strains was performed. **A** VP1 and **B** VP2 epitope sequence conservation was analyzed in different genotypes of norovirus. Representative strains, mutant strains and recombinant strains of genotypes GII.4, GII.3, and GII.2 were selected for comparison. Each letter represents an amino acid residue, and the size of the letter reflects the conservation of this site

The GII.4 Sydney 2012 [P16] used in this study has become an epidemic genotype in recent years. Meanwhile, according to the phylogenetic tree of the VP1 and VP2 sequence (Additional file 1: Fig. S3, S4), it can be found that GII.4 Sydney 2012 [P16] strain had very little genetic variation with each GII.4 mutant or recombinant strain, and had a small evolutionary distance with other GII strains. Thus, it can be used as a representative of GII genotype norovirus to carry out research. Sequence similarity analysis of the above epitopes was carried out, and the comparison objects were representative strains, mutant strains and recombinant strains of GII.4, GII.3, and GII.2 of the top three epidemic strains. VP1_199–216_ was found to be the most conserved epitope (Fig. 4A), which was completely consistent among all strains of GII.4, and had a percent sequence similarity of more than 88% with GII.3 and GII.2 (data not shown). The other epitopes also had small changes in site, and all of them are conserved among common strains (Fig. 4A, B).

### VP1_199–216_- and VP1_469–492_-specific antibodies partially block VLP binding to the HBGA receptor

**Fig. 5:**
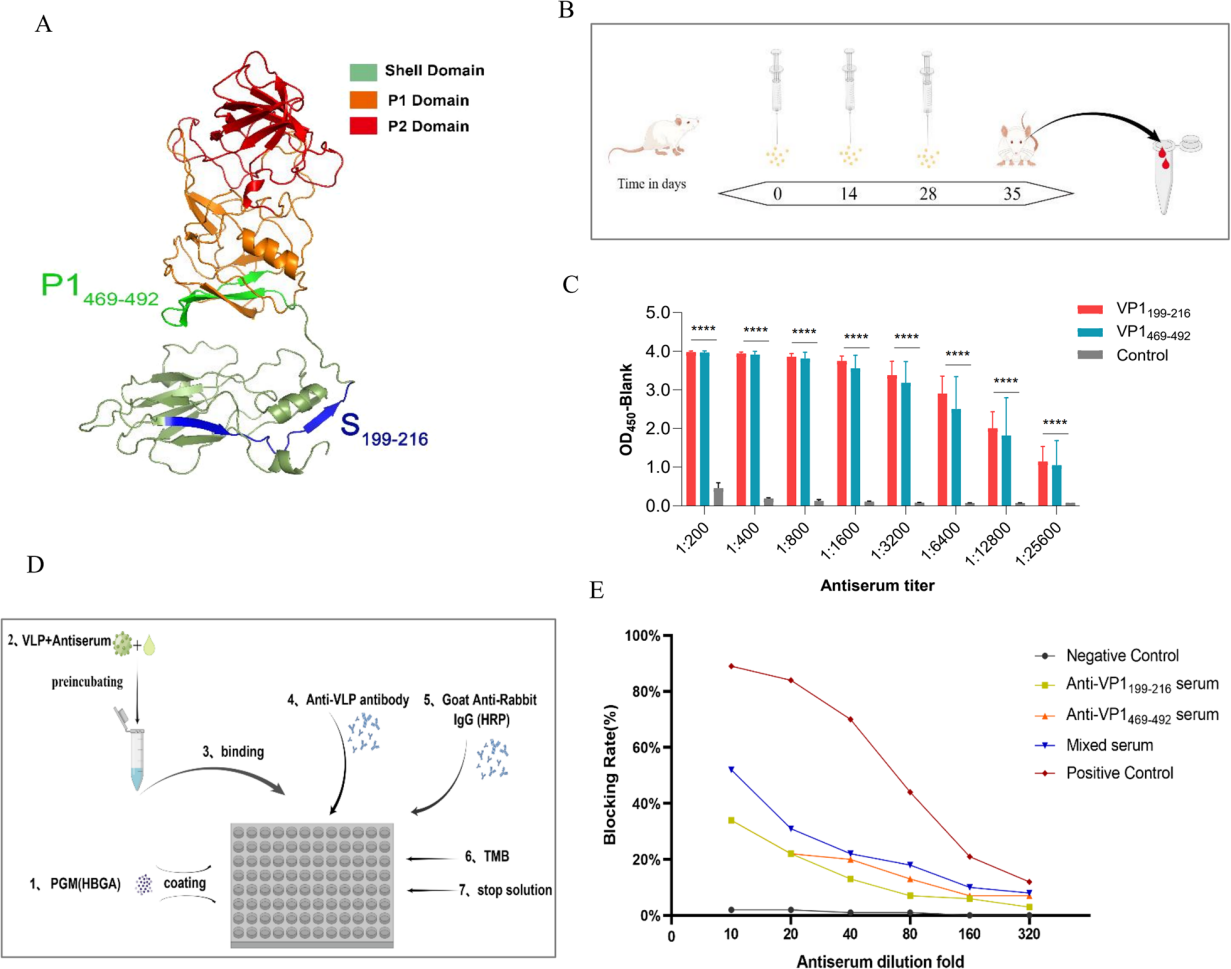
VLP-HBGA binding antibody blockade assay. **A** Spatial structure of the VP1 protein and locations of linear epitopes. **B** Schematic of immunizing mice with epitope-KLH conjugation by Figdraw. **C** The titer of antiserum was detected by peptide ELISA. The control group was normal mouse serum immunized with NaCl and adjuvant. **D** Diagram of VLP-carbohydrate binding blockade assay by Figdraw. **E** The blocking efficiency of the antiserum, with normal mouse serum as the negative control and the Anti-Norovirus GII.4 (Absolute Antibody, NVB43.9) which have a block function as the positive control; mixed serum is a mixture of two epitope antisera

Subsequently, the three-dimensional structure of the VP1 protein was constructed to locate the epitope, it can be seen that VP1_199–216_ and VP1_469–492_ epitopes were located in the S and P1 domains, respectively, far from the HBGA binding pocket of the P2 domain (Fig. 5A). In order to explore whether VP1 epitope-specific antibodies can block norovirus binding to HBGA, receptor blocking tests were conducted. First, BALB/c mice were immunized subcutaneously with peptide-conjugated KLH plus adjuvant at 0, 14 and 28 days to obtain epitope-specific antiserum (Fig. 5B). The results of the peptide-ELISA test proved that the OD value of the antiserum was significantly higher than that of the control until the dilution was 1:25,600, indicating that the preparation of the antiserum may be successful. (Fig. 5C). Additionally, we successfully expressed VLPs in the insect expression system and purified them (Additional file 1: Fig. S1C, D). The process of the blocking assay is shown here (Fig. 5D). To quantify the blocking effect of antiserum, we calculated the blocking rate with a serum-free well as a reference. When the concentration of antiserum was 1:10, the blocking rate of both VP1_199–216_ and VP1_469–492_ antiserum was 34%, and that of the mixed serum reached 52% (Fig. 5E). Although these antisera were not as effective as the positive control, their effect significantly higher than that of the negative control, with a moderate blocking effect.

## Discussion

This study mapped the specific IgG response profiles of VP1 and VP2 in individuals with previous NoV infection and determined linear antigenic B-cell epitopes that elicited strong and universal IgG responses. Furthermore, VP1_199–216_ and VP1_469–492_ epitope-specific antiserum showed a moderate ability to block VLP binding to cell receptors. It could enhance the understanding of norovirus capsid protein-specific IgG responses and reveal some protective mechanisms of antibodies, providing insights into vaccine development.

According to our results, the overall NoV-specific (VP1 and/or VP2) IgG-positive rate was approximately 94%, which may be slightly different from what has been reported. Previous studies have shown that the seroprevalence of GII-NoV varies by region, antigen and detection method. For example, the seropositivity rates in Pelosi, Honma and Mesquita’s studies were 91.2%, 86.7% and 70%, respectively [34–36].

For a long time, there have been few studies on VP2, but some of them have shown that VP2 is essential for the stable expression and nuclear localization of VP1 [11, 37] and may regulate the antigen presentation of B cells to modulate immune responses [38]. Our study found, for the first time, strong VP2-specific IgG responses in past norovirus-infected individuals, although they were significantly weaker than the VP1-specific IgG response. There are two main possible reasons for the significant differences in IgG response levels between VP1 and VP2. First, the VP1 protein is more abundant in viruses than the VP2 protein, with each virion containing 180 VP1 proteins and only 1–8 VP2 proteins [39, 40]. Second, VP1 is more easily exposed to elicit an immune response than VP2, with VP1 forming the outer viral capsid and VP2 being inside VP1 [39]. In addition, differences in immunogenicity may also be another influencing factor, but this is not yet clear. A previous study mentioned that adding the VP2 sequence to the C-terminal or N-terminal of VP1 does not change its antigenicity in VLPs, which possibly suggested that the antigenicity of VP2 is lower than that of VP1 and may even be negligible [41]. Another report discovered high levels of VP2-specific T cells isolated from seropositive individuals, but slightly lower than VP1 [42]. In short, the immunity and function of VP2 are not entirely clear and need to be explored deeply in the future.

In contrast to VP2, many B-cell epitopes on VP1 have been reported, some of which overlap with epitopes identified in this study at some sites [23]. A mouse monoclonal antibody 2B3mAb (recognizing amino acid sites 181–220 of VP1) and two porcine monoclonal antibodies (recognizing sites 1–216 of VP1) had overlapping sites with the VP1_199–216_ epitope, and they recognized multiple GII genotypes of NoV, showing great potential as reagents for NoV detection [33, 43]. In addition, multiple reported conformational or linear epitopes covered partial amino acid sites of the VP1_469–492_ epitope. HJT-R3-F7 antibody (recognizing site 473–488) could effectively detect VLP antigen and virions of clinical samples and had the potential to be used as a diagnostic reagent [44]. The Nano-26 antibody (overlapping sites 470–472, 475) blocked the binding of VLPs to HBGA and cross-binded with multiple genotypes of NoV, so it may be used as a novel therapeutic agent against NoV [45]. NORO-320 IgA (overlapping site 478, 481–482, 484, 486) had extensive cross-reactivity with GII-NoV and had receptor-blocking and viral neutralization ability, which could potentially be used to develop a broadly protective NoV vaccine [46]. Therefore, VP1_199-216_ and VP1_469-492_ epitopes may be of great value in the development of diagnostic reagents, anti-NoV therapeutics and vaccines.

In this study, VP1 epitopes were far from the HBGA binding pocket, so the blocking effect of epitope-specific antibodies was not mediated by direct competition with the HBGA binding site, presenting a moderate blocking effect. The blocking antibodies mentioned above could help explain the blocking mechanism in this study. Among them, NORO-320 IgA exerted a blocking role by polyvalent antibody cross-linking to form steric hindrance, and the recombinant IgG form of NORO-320 also had blocking ability, while the recombinant Fab form may not have blocking function due to its small molecular weight, which cannot lead to steric hindrance [46]. In addition, the Nano26 antibody targeted sites far away from the HBGA binding pocket, and capsid deformation and degradation were observed after treatment of VLPs with the antibody. Therefore, it acted as blocking function by disrupting capsid morphology [29, 45]. In summary, there are two possible mechanisms for the blocking effect in this study. One is that the polyvalent antibody cross-linking formed steric hindrance to block binding to the HBGA receptor, and the other is that the binding of the antibody to VLPs caused morphological changes that interfered with the binding of the HBGA receptor. However, the specific mechanism needs to be verified by further experiments.

Rapid mutation of norovirus, low immune persistence and weak cross-protection contribute to repeated infection [22, 47, 48]. At present, most of the known blocking epitopes are located in the highly variable P2 domain and cannot cross-protect multiple genotypes of NoV infection [23, 48]. Although the epitopes in this study were conserved and universal and had a modest blocking function, they could not exert a good protective effect alone. Because the epitope-specific response was not found in the whole population and their blocking effect was relatively weak, the protection rate may be low if applied to the vaccine alone, and the combination of multiple epitopes was needed to achieve full protection. It is suggested that the multi-epitope strategy for vaccine or therapeutic agent development may achieve broad and persistent protection, or perhaps produce specific vaccines for populations with different antibody response patterns.

There are still some limitations in this study. First, we did not collect current NoV-infected serum samples, so the value of epitopes in the diagnosis of infection and its effect on disease remains unclear. Second, we did not explore the function of the VP2-specific epitopes, which should be investigated in our future research program.

In summary, we used VP1 and VP2 protein ELISA and peptide ELISA methods to generate a NoV-specific IgG antibody response profile in past NoV-infected individuals, determined linear blockade epitopes on VP1, and identified linear antigenic B-cell epitopes of VP2 for the first time. Our findings contribute to a better understanding of the specific IgG response to the NoV capsid protein and may aid in the development of a vaccine.

## Supplementary Information

**Additional file 1.** Supplementary Figure and Supplementary Table. **Figure S1.** Purified VP1, VP2 protein and VLP. **Figure S2.** VP1 and VP2 specific IgG response were compared in different sex and age groups. **Figure S3.** Phylogenetic tree based on VP1 sequence. **Figure S4.** Phylogenetic tree based on VP2 sequence. **Table S1.** The composition of all subjects. **Table S2.** All peptides of VP1 and each POOL. **Table S3.** All peptides of VP2 and each POOL.

## Data Availability

Data are available on reasonable request.

## Abbreviations

NoV: Norovirus
VLPs: Virus-like particles
HBGA: Histo-blood group antigen
HR: High responses
TR: Total responses
