# Supplementary material for: Linear epitopes on the capsid protein of norovirus commonly elicit high antibody response among past-infected individuals
Source: Virol J. 2023 Jun 6;20:115. doi: 10.1186/s12985-023-02087-y (PMC10242594; doi:10.1186/s12985-023-02087-y)
Supplement: Supplementary file 1 — Additional file 1. Supplementary Figure and Supplementary Table. Figure S1. Purified VP1, VP2 protein and VLP. Figure S2. VP1 and VP2 specific IgG response were compared in different sex and age groups. Figure S3. Phylogenetic tree based on VP1 sequence. Figure S4. Phylogenetic tree based on VP2 sequence. Table S1. The composition of all subjects. Table S2. All peptides of VP1 and each POOL. Table S3. All peptides of VP2 and each POOL. [file 12985_2023_2087_MOESM1_ESM.docx]

**Supplementary Figure**

**
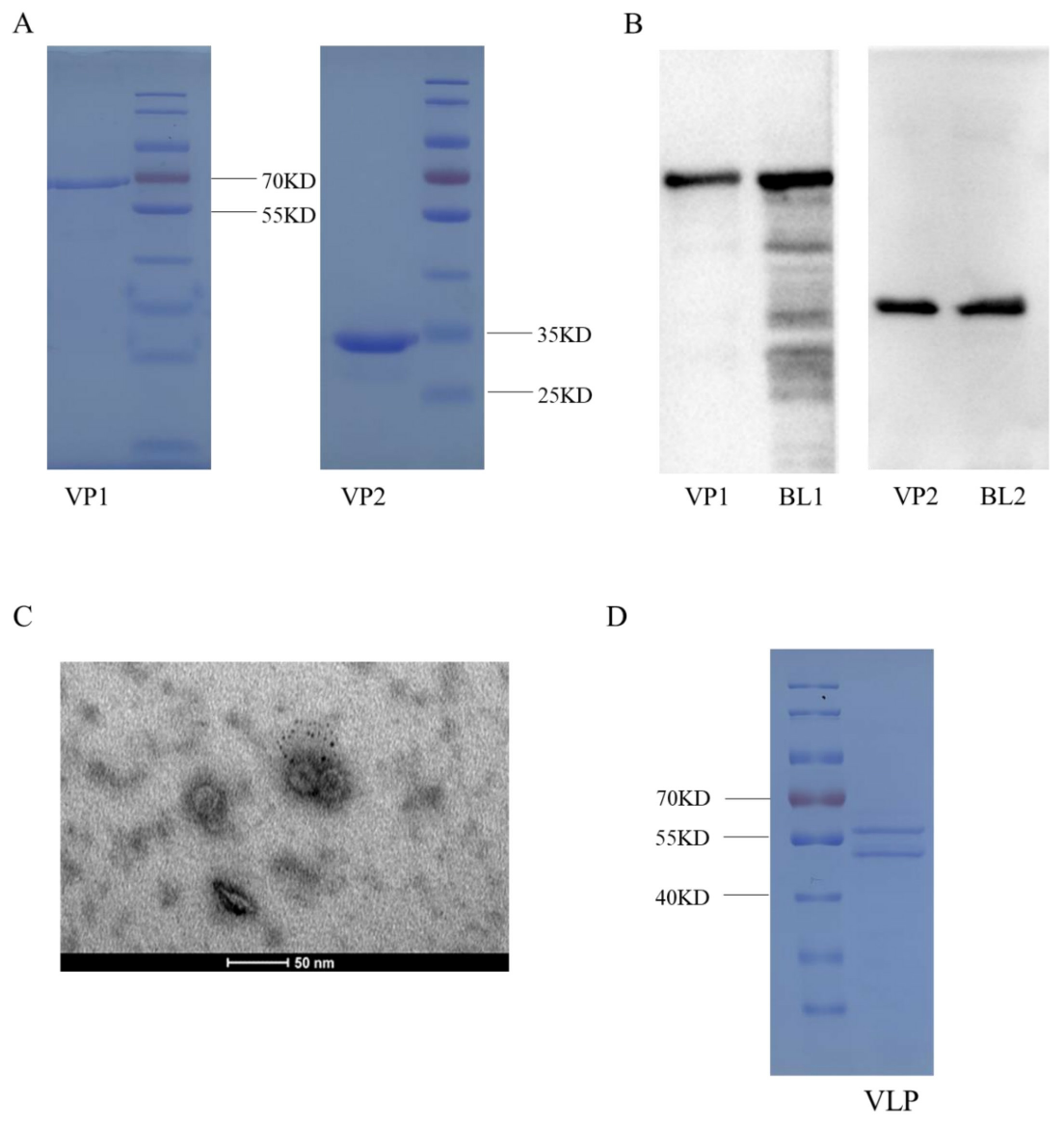
**

**Figure S1. Purified VP1 , VP2 protein and VLP**

(A) The purified VP1 and VP2 protein were stained by commassie blue staining after SDS-PAGE. (B) Western blot results of purified VP1 and VP2 protein. BL_1_: Bacterial lysate expressing VP1 protein; BL_2_: Bacterial lysate expressing VP2 protein. (C) Transmission electron microscope image of VLP after negative staining. (D) The purified VLP were stained by commassie blue staining after SDS-PAGE.

**
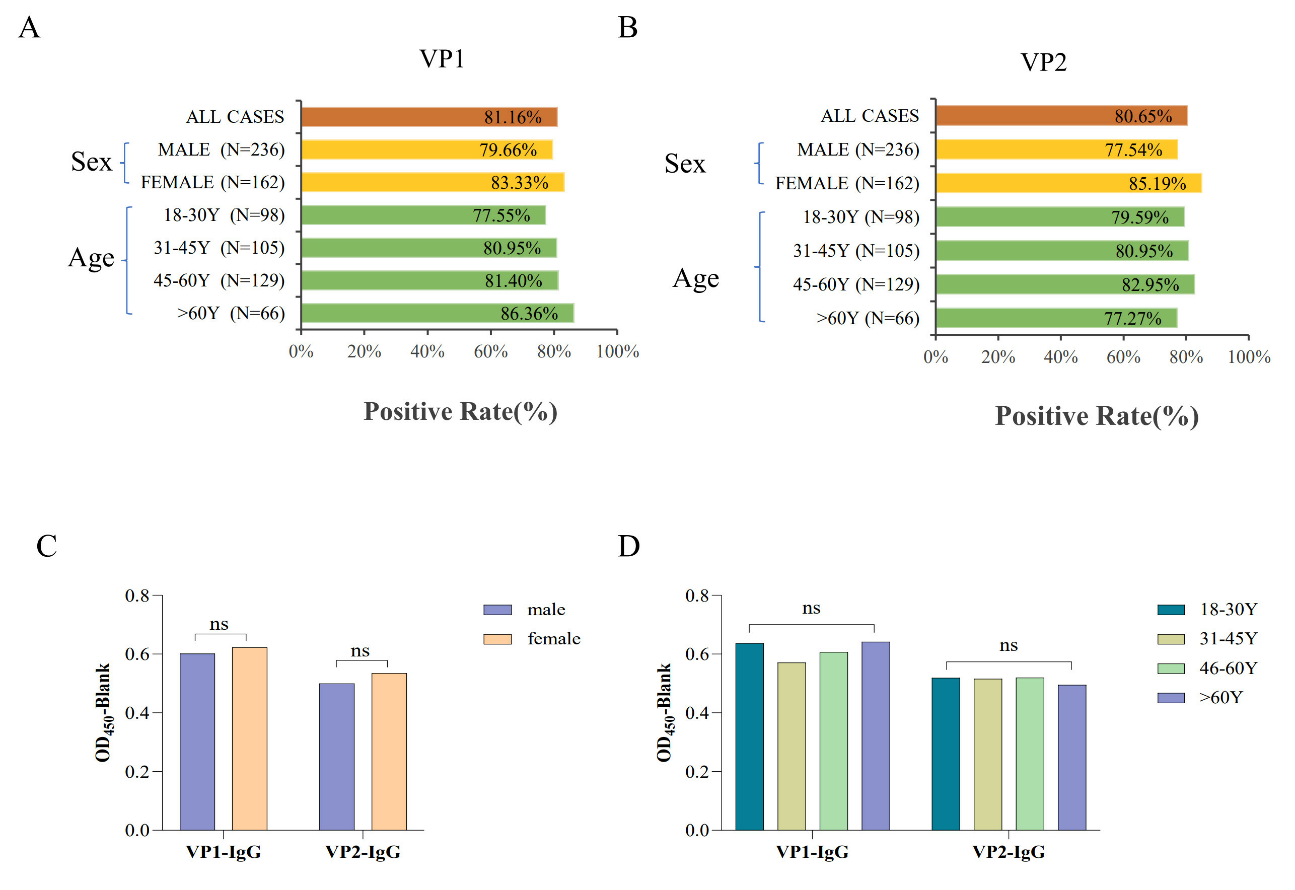
**

**Figure S2. VP1 and VP2 specific IgG response were compared in different sex and age groups.**

(A)VP1-specific IgG (B) VP2-specific IgG positive rate were compared among different sex,age groups. (C) VP1 and VP2 specific IgG level were compared among different sex groups. (D) VP1 and VP2 specific IgG level were compared among different age groups.

**
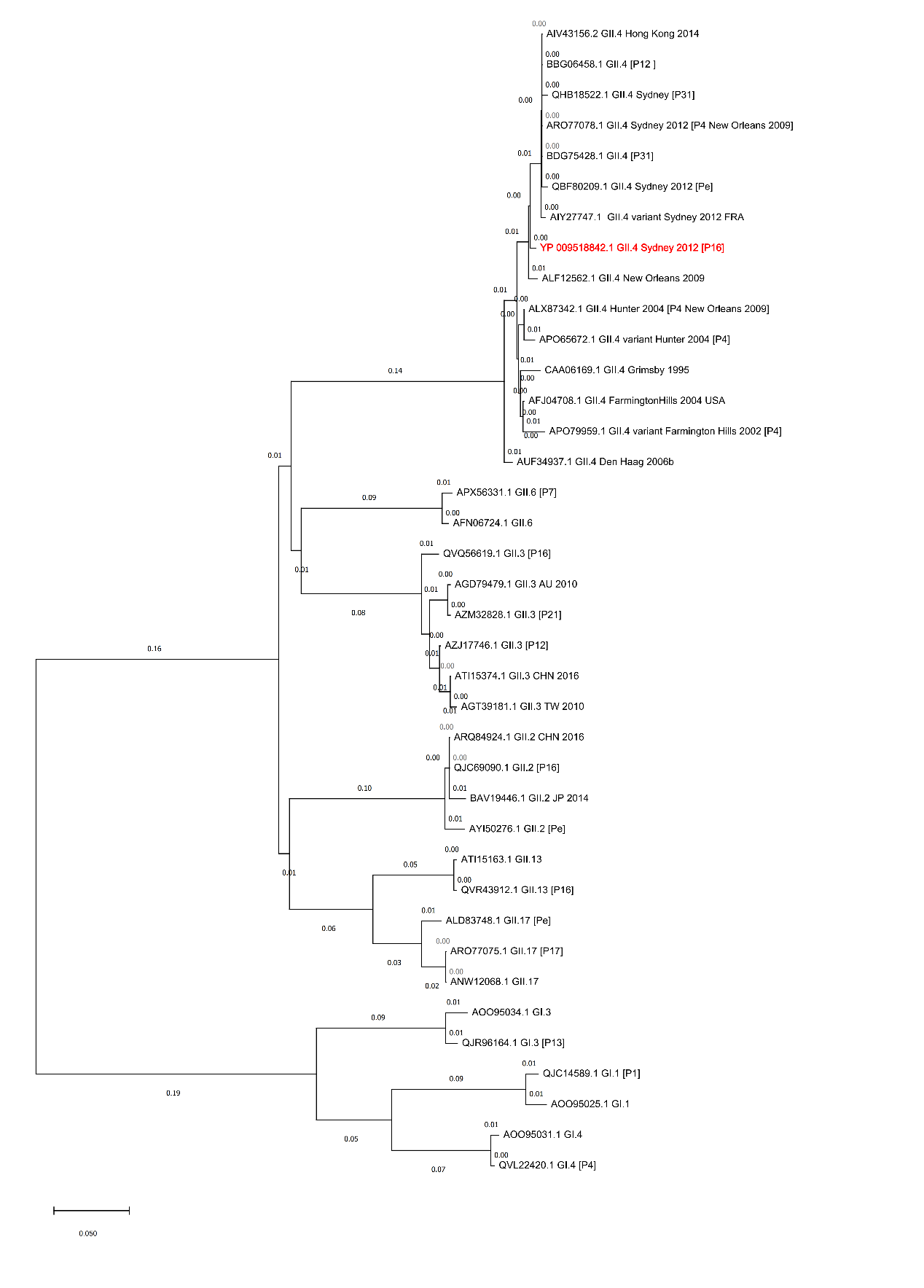
**

**Figure S3. Phylogenetic tree based on VP1 sequence.**

**
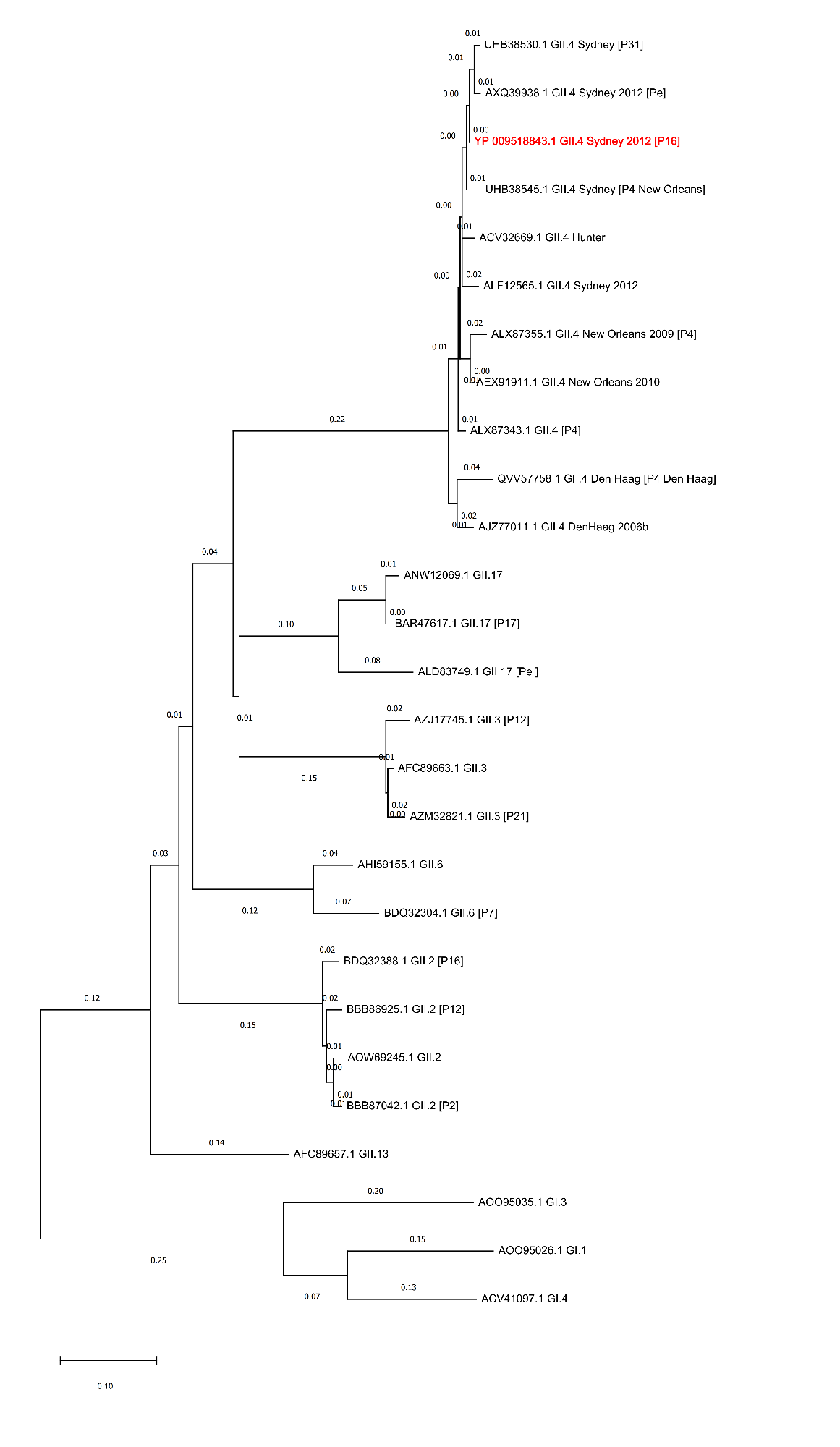
**

**Figure S4. Phylogenetic tree based on VP2 sequence.**

**Supplementary Table**

**Table S1. The composition of all subjects.**

| Total individuals | Gender | | Age | | | |
| --- | --- | --- | --- | --- | --- | --- |
|  | male | female | 18-30y | 31-45y | 46-60y | 61y- |
| 398 | 236 | 162 | 98 | 105 | 129 | 66 |

**Table S2. All peptides of VP1 and each POOL.**

| POOL | | | | | VP1（540aa）- Peptides | | | | | |
| --- | --- | --- | --- | --- | --- | --- | --- | --- | --- | --- |
| POOL1 | 1-18 | 7-24 | 13-30 | 19-36 | 25-42 | 31-48 | 37-54 | 43-60 | 49-66 | 55-72 |
| POOL2 | 61-78 | 67-84 | 73-90 | 79-96 | 85-102 | 91-108 | 97-114 | 103-120 | 109-126 | 115-132 |
| POOL3 | 121-138 | 127-144 | 133-150 | 139-156 | 145-162 | 151-168 | 157-174 | 163-180 | 169-186 | 175-192 |
| POOL4 | 181-198 | 187-204 | 193-210 | 199-216 | 205-222 | 211-228 | 217-234 | 223-240 | 229-246 | 235-252 |
| POOL5 | 241-258 | 247-264 | 253-270 | 259-276 | 265-282 | 271-288 | 277-294 | 283-300 | 289-306 | 295-312 |
| POOL6 | 301-318 | 307-324 | 313-330 | 319-336 | 325-342 | 331-348 | 337-354 | 343-360 | 349-366 | 355-372 |
| POOL7 | 361-378 | 367-384 | 373-390 | 379-396 | 385-402 | 391-408 | 397-414 | 403-420 | 409-426 | 415-432 |
| POOL8 | 421-438 | 427-444 | 433-450 | 439-456 | 445-462 | 451-468 | 457-474 | 463-480 | 469-486 | 475-492 |
| POOL9 | 481-498 | 487-504 | 493-510 | 499-516 | 505-522 | 511-528 | 517-534 | 523-540 |  |  |

**Table S3. All peptides of VP2 and each POOL.**

| POOL | VP2（268aa）- Peptide | | | | |
| --- | --- | --- | --- | --- | --- |
| POOL1 | 1-18 | 7-24 | 13-30 | 19-36 | 25-42 |
| POOL2 | 31-48 | 37-54 | 43-60 | 49-66 | 55-72 |
| POOL3 | 61-78 | 67-84 | 73-90 | 79-96 | 85-102 |
| POOL4 | 91-108 | 97-114 | 103-120 | 109-126 | 115-132 |
| POOL5 | 121-138 | 127-144 | 133-150 | 139-156 | 145-162 |
| POOL6 | 151-168 | 157-174 | 163-180 | 169-186 | 175-192 |
| POOL7 | 181-198 | 187-204 | 193-210 | 199-216 | 205-222 |
| POOL8 | 211-228 | 217-234 | 223-240 | 229-246 | 235-252 |
| POOL9 | 241-258 | 247-264 | 253-268 |  |  |
